# Biliverdin targets enolase and eukaryotic initiation factor 2 (eIF2α) to reduce the growth of intraerythrocytic development of the malaria parasite *Plasmodium falciparum*

**DOI:** 10.1038/srep22093

**Published:** 2016-02-26

**Authors:** Eduardo Alves, Fernando V. Maluf, Vânia B. Bueno, Rafael V. C. Guido, Glaucius Oliva, Maneesh Singh, Pedro Scarpelli, Fahyme Costa, Robson Sartorello, Luiz H. Catalani, Declan Brady, Rita Tewari, Celia R. S. Garcia

**Affiliations:** 1 Núcleo de Pesquisa em Sinalização Celular Patógeno-Hospedeiro (NUSCEP), Departamento de Fisiologia, Instituto de Biociências, Universidade de São Paulo, Brasil; 2 Centro de Pesquisa e Inovação em Biodiversidade e Fármacos, Instituto de Física de São Carlos, Universidade de São Paulo, Brasil; 3 Departamento de Química Fundamental, Instituto de Química, Universidade de São Paulo, Brasil; 4 Departamento de Parasitologia, Instituto de Ciências Biomédicas, Universidade de São Paulo, Brasil; 5 School of Life Sciences, University of Nottingham, UK

## Abstract

In mammals, haem degradation to biliverdin (BV) through the action of haem oxygenase (HO) is a critical step in haem metabolism. The malaria parasite converts haem into the chemically inert haemozoin to avoid toxicity. We discovered that the knock-out of HO in *P. berghei* is lethal; therefore, we investigated the function of biliverdin (BV) and haem in the parasite. Addition of external BV and haem to *P. falciparum*-infected red blood cell (RBC) cultures delays the progression of parasite development. The search for a BV molecular target within the parasites identified *P. falciparum* enolase *(Pf* enolase) as the strongest candidate. Isothermal titration calorimetry using recombinant full-length *Plasmodium* enolase suggested one binding site for BV. Kinetic assays revealed that BV is a non-competitive inhibitor. We employed molecular modelling studies to predict the new binding site as well as the binding mode of BV to *P. falciparum* enolase. Furthermore, addition of BV and haem targets the phosphorylation of *Plasmodium falciparum* eIF2α factor, an eukaryotic initiation factor phosphorylated by eIF2α kinases under stress conditions. We propose that BV targets enolase to reduce parasite glycolysis rates and changes the eIF2α phosphorylation pattern as a molecular mechanism for its action.

The expansion of malaria intervention has had a tremendous impact on malaria incidence and mortality worldwide; however, the lack of a commercial vaccine and the increase in drug-resistant strains highlight the importance of identifying new mechanisms to combat the disease. Malaria symptoms appear when red blood cell (RBC) infection occurs and follows a series of developmental stages known as ring (R), trophozoite (T) and schizont (S). After cell lysis, the invasive form merozoites are released and subsequently invade other RBCs to reinitiate the cycle^1^,^2^. During the intraerythrocytic phase, the host haemoglobin is degraded and used as a source of amino acids^3^. This process generates haem, a toxic molecule that is scavenged by the malaria parasite through the formation of haemozoin polymer^4^.

Most mammalian cells use a different strategy to nullify haem toxicity by converting it to biliverdin (BV), a step catalysed by haem oxygenase (HO). BV is subsequently converted to bilirubin (BR) by biliverdin reductase (BVR)^5^,^6^. In the past, BV and its by-product BR have been exclusively viewed as waste products of haem catabolism^7^. However, there are indications that haem, BV and BR may play important roles in gene expression, oxidative response, and cellular signal transduction pathways in several biological systems^8^,^9^,^10^. Haem is a prosthetic group indispensable for the growth of malaria parasites^11^,^12^,^13^. Recently, it has been demonstrated that *P. falciparum* possess a *de novo* haem biosynthetic pathway^6^. The existence of a haem oxygenase enzyme in *P. falciparum* (*Pf*HO) was demonstrated^14^,^15^, however, its activity is controversial^16^,^17^. Therefore, the role of *Plasmodium* HO in the haem metabolism and the impact of host BV on host-parasite interaction remain unclear.

Phosphorylation is among the most important post-translational modifications for eukaryotic cells as well for *Plasmodium*^18^. In general, the kinases that regulate the eukaryote translation of proteins in response to stress are called eukaryotic initiation factor 2α kinases (eIF2α kinases), and the mechanism of action involves changes in the phosphorylation of eIF2α under stress conditions, which inhibits the translation of most proteins. In *Plasmodium,* three eIF2α kinases were identified: PfeIK1, PfeIK2 and PfPK4^18^. PfeIK1 and PfPK4 are more abundant in asexual stages, whereas PfeIK2 is more abundant in sporozoites^19^. A.P. Han *et al.* 2001^20^ identified the presence of an eIF2α kinase in reticulocytes whose action is modulated by haem. The three-dimensional structure has two haem-binding sites that suppress the phosphorylation activity of eIF2α. Thus, eIF2α kinase HRI (haem-regulated inhibitor) prevents the development of reticulocytes in the absence of haem through the phosphorylation of eIF2α^21^. Here, we investigated changes in the phosphorylation pattern of *Plasmodium* eIF2α factor upon the addition of BV and haem into *P. falciparum*-infected RBCs.

To the best of our knowledge, this work is the first to investigate the effect of BV on intraerythrocytic development and its potential targets in *P. falciparum*. We demonstrate that BV is present in infected RBC cultures. The higher BV concentration affects the *P. falciparum* cell cycle by increasing the abundance of early stages and reducing late stages. We identify enolase as a target for BV binding in *P. falciparum* and evaluate the stoichiometry and energetic components underlying BV binding. Moreover, our data indicate BV as a non-competitive inhibitor at the low micromolar range, and molecular modelling suggests a BV-binding mode for *P. falciparum* enolase.

## Results

### Detection of BV in *P. falciparum*–infected red blood cell supernatant culture

**Figure 1:**
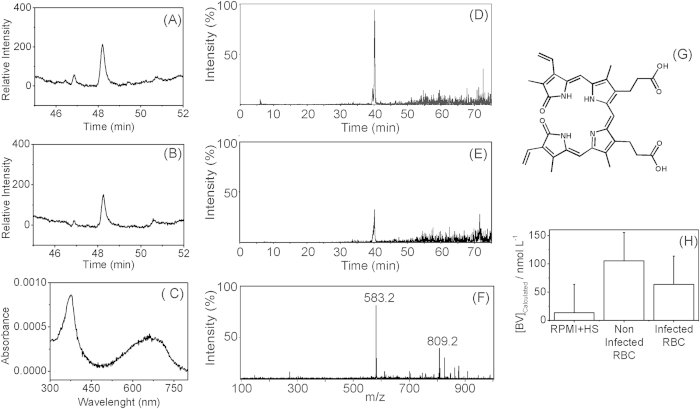
BV characterization in *P. falciparum* culture and non-infected supernatant. **(A**,**B**) are chromatographic analysis at 660 nm of uninfected and infected culture supernatants, respectively. (**C**) is the absorption spectra observed for (**A**) at 48.3 min retention time. (**D**,**E**) are ESI + MS chromatographic analysis (EIC m/z 583) of non-infected and infected culture supernatants, respectively. (**F**) is the mass spectrum observed for **(D**) at 40 min retention time. Different RTs are due to changes in the mobile phase necessary for LC-MS. BV structure (**G**) presents a calculated monoisotopic mass of 582.2 Da. Quantitative analysis (**H**) reveals similar concentrations of BV in infected and non-infected culture supernatants.

To investigate the host BV in *Plasmodium* culture, the amounts of BV in the supernatant of uninfected and *P. falciparum-*infected RBC cultures were measured by high-performance liquid chromatography (HPLC-UV-Vis and HPLC-MS; Fig. 1). Both uninfected (Fig. A) and infected (Fig. 1B) RBC culture supernatant chromatograms exhibited a UV-Vis peak at 48.3 min due to BV. ESI-MS analysis of the eluate exhibited a strong peak at m/z 583.2 (Fig. 1D,E), corresponding to the molecular ion of HBV^+^ (protonated BV). BV quantification, which was performed by the addition of external standards, revealed no significant difference between BV concentration in supernatant samples obtained from uninfected and infected RBC cultures (105 ± 30 and 63 ± 30 nM, respectively; Fig. 1H).

### Effect of BV in *P. falciparum* cell cycle and *P. berghei* haem oxygenase (*Pb*OH) knockout

**Figure 2:**
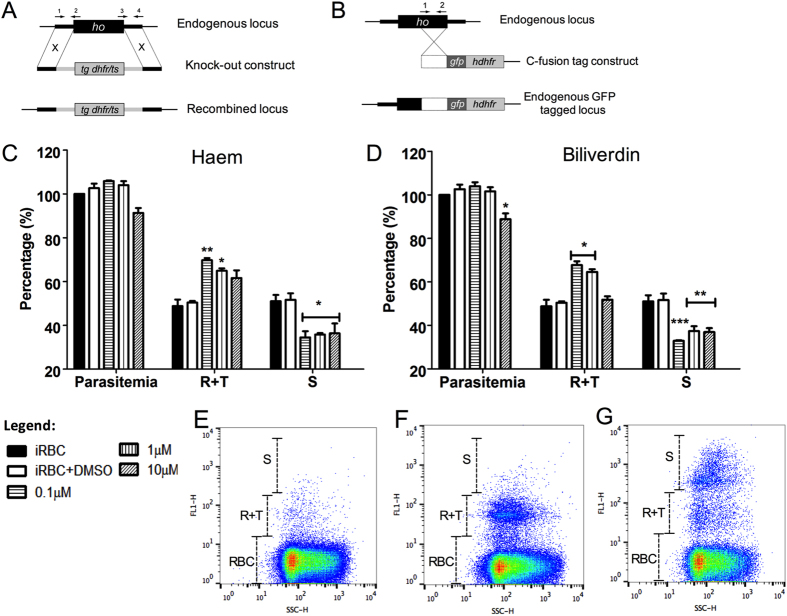
Multiple strategies to investigate the function of haem oxygenase and effects of haem and biliverdin during erythrocytic development of *Plasmodium*. (**A**) Schematic representation of the gene targeting strategy used for gene disruption via double homologous recombination in *P. berghei*. Primers 1 to 4 used for PCR of 5′ and 3′ fragments for knockout construct are indicated. (**B**) Schematic representation of the gene-tagging construct used for tagging of the endogenous *pbho* locus in *P. berghei* by single homologous recombination with *gfp*. Primers 1 and 2 used for amplifying the endogenous locus are indicated. (**C**) *P. falciparum* intraerythrocytic modulation during 48 hours of incubation with haem. Parasitaemia value of the control with no drugs or solvent (iRBC) was considered 100%. All treatments were compared with the control containing only solvent (iRBC + DMSO). iRBC + DMSO values: parasitaemia 103 ± 2%; R + T: 50 ± 1% and S: 52 ± 5%. Haem graphic, parasitaemia values: 0.1 μM (99 ± 3%); 1 μM (97 ± 5%); 10 μM (91 ± 2%). R + T values: 0.1 μM (70 ± 1%); 1 μM (64 ± 1%); 10 μM (62 ± 4%). S values: 0.1 μM (35 ± 3%); 1 μM (36 ± 1%); 10 μM (36 ± 4%). (**D**) BV graphic, parasitaemia values: 0.1 μM (104 ± 2%); 1 μM (98 ± 4%); 10 μM (89 ± 3%). R + T values: 0.1 μM (68 ± 1%); 1 μM (65 ± 1%); 10 μM (52 ± 2%). S values: 0.1 μM (33 ± 1%); 1 μM (37 ± 2%); 10 μM (37 ± 2%). The graphs in C and D represent means and standard error of three independent experiments. *P < 0.05%, **P < 0.01% and ***P < 0.001%. (**E**) Dot plot depicts a typical gating population of non-infected RBCs (RBC). (**F**) Dot plot depicts a typical gating population of RBCs and infected RBCs with mononucleated parasites (R + T). (**G**) Dot plot depicts a typical gating population of RBCs, R + T and infected RBCs with multinucleated parasites (S).

An alternative strategy to deplete the levels of haem within cells relies on the activity of haem oxygenase enzymes, which convert haem into BV, iron, and carbon monoxide. *P. falciparum* possess a haem oxygenase (PfHO)^14^,^15^; however, its cellular function within parasites remains unclear^17^. Therefore, to investigate the role of HO in *Plasmodium*, a strategy of knocking out the *P. berghei* haem oxygenase (PbHO) (Fig. 2A) and adding c-fusion GFP (green fluorescence protein) to PbHO (Fig. 2B) was performed. Both approaches were lethal to *P. berghei*, suggesting an unknown vital role for this enzyme in *Plasmodium* physiology.

Moreover, the detection of BV at nanomolar concentrations in parasite culture led to the investigation of BV as a signal modulator during the *P. falciparum* life cycle forms R, T and S. For this purpose, synchronized infected RBCs in the ring stage were incubated for 48 hours in the absence and presence of 0.1, 1 and 10 μM of haem and BV (Fig. 2C,D, respectively). The distribution of *P. falciparum* in the R, T, and S forms was evaluated by counting 10^5^ cells (Fig. E–G). Here, 10 μM BV exhibited a toxic effect after 48 hours (Fig. 2D). In addition, 0.1 and 1 μM BV and haem did not change the parasitaemia after 48 hours of incubation compared with the controls; however, at these concentrations, both compounds increase the R + T forms and decrease the S form.

### Identification of BV targets in *P. falciparum*

**Figure 3:**
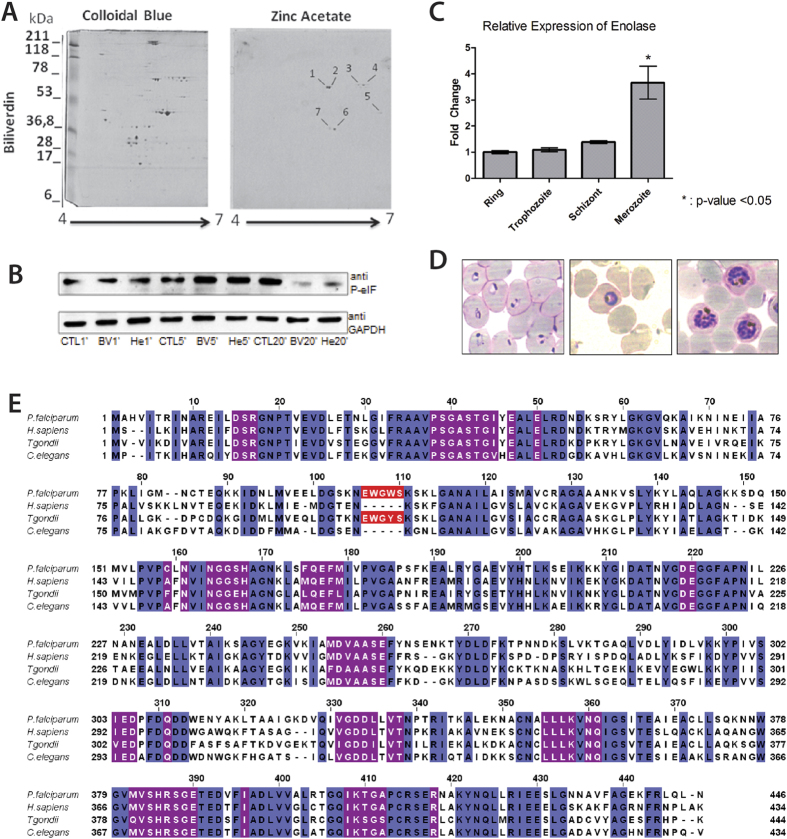
BV binding protein assay in two-dimensional electrophoresis, phosphorylation of eIF2α, relative expression of enolase gene throughout 3D7 strain intraerythrocytic cycle, and enolase amino acid alignment. (**A**) Total *P. falciparum* protein extract pre-treated with 20 μM BV for 30 minutes, revealing total protein staining (left colloidal Coomassie). On the right, the same gel reveals 7 positive spots (indicated by black lines) on zinc acetate assay treatment. (**B**) Western blot using antibody anti-phospho eIF2α (anti P-eIF) in *P. falciparum*-infected RBCs at trophozoite stage incubated with BV (BV) and haem (He) for 1, 5 or 20 minutes. GAPDH was used as a loading control. (**C**) Parasites were synchronized to isolate ring, trophozoite, schizont and merozoite stages using sorbitol. RNA samples were extracted, purified and subjected to qRT-PCR. Seryl-tRNA synthetase expression was quantified to normalize mRNA levels. The graphics reveal an increase in enolase expression in the merozoite stage. ^★^Statistically significant by t-test with P < 0.05. (**D**) Blood smears of *P. falciparum* culture indicating the stages (ring, trophozoite, schizont, respectively, from left to right) used to extract RNA for qRT-PCR analysis. (**E**) Amino acid alignment of enolase sequences of *H. sapiens*, *P. falciparum*, *T. gondii* and *C. elegans*. Identical residues are highlighted in blue, the pentapeptide that differentiates *P. falciparum* enolase from other organisms in red, and the residues that compose the catalytic site of the enzyme are indicated in purple.

To investigate the mechanism of BV modulation in the *P. falciparum* cell cycle, a binding protein-BV assay was performed to identify candidate BV molecular target proteins (Fig. 3A). Zinc acetate was used to reveal protein-BV interactions, as described in Berkelman and Lagarias^22^. The results of mass spectrometry (MS) analysis of zinc acetate/Coomassie staining are shown in Table S1 in the supplemental material. The protein-BV binding assay using zinc acetate in a two-dimensional gel revealed seven positive spots (Fig. 3A) containing *P. falciparum* proteins (Table S1). As the number of peptides for enolase (access number: Pf10_0155) is considerably increased compared with the remaining peptides, we concluded that the BV-binding assay revealed *P. falciparum* enolase as the strongest candidate for a BV molecular target. Moreover, we have investigated the phosphorylation of eIF2α eukaryotic initiation factor as an indicator of stress factor for the cell after incubating *P. falciparum*-infected RBC at trophzoite stage with 1 μM haem and 1 μM BV by 1, 5 and 10 minutes followed by Western blot analysis using antibody anti-phospho eIF2α (anti P-eIF) (Fig. 3B). The results revealed that BV and haem promotes changes in the phosphorylation pattern of eIF2α and this could be part of their mechanism for reducing the growth of intraerythrocytic development of the malaria parasite *Plasmodium falciparum.*

Additionally, we have analysed changes in enolase expression throughout *P. falciparum* intraeritrocytic lifecycle at R, T and S stages. We detected an increase in enolase expression on merozoites, the RBC invasive form, (Fig. 3C). Fig. 3D illustrates R, T and S phases of the parasite lifecycle. Amino acid alignment with enolases from *P. falciparum* with other organisms (Fig. 3E) reveals that the enzyme has a peptide that is conserved only in apicomplexa phylum.

### Recombinant purified *Pf* enolase-BV binding proprieties and *in silico* docking of BV-*Pf* enolase

**Figure 4:**
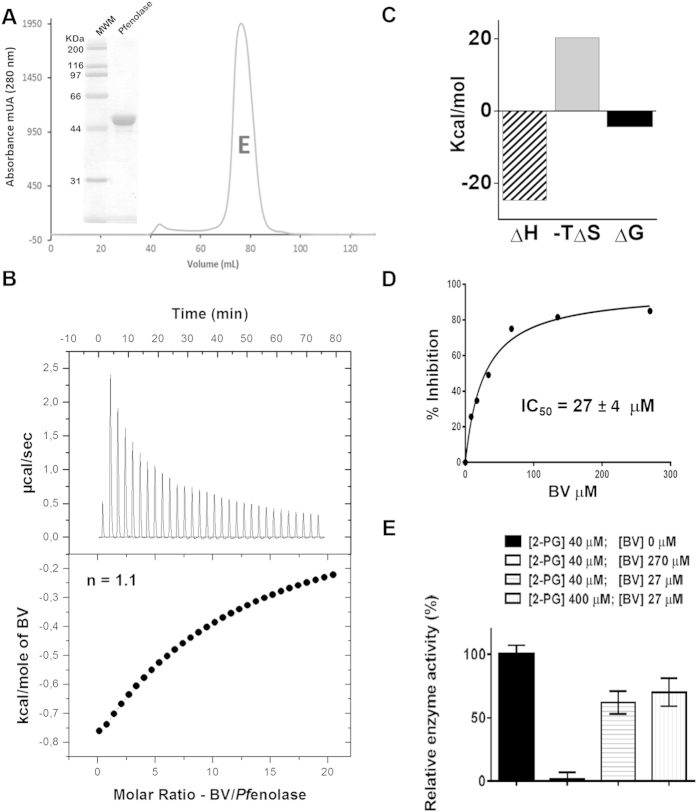
BV binding properties and inhibitory activity on Pf enolase. (**A**) SDS–PAGE (10%) and gel filtration chromatogram of purified recombinant *Pf* enolase (MWM = molecular weight mass, E = enolase). (**B**) Isothermal titration calorimetry measurement of BV binding to *Pf* enolase. Top panel: changes in heat over time as BV is titrated into *Pf* enolase. Bottom panel: normalized change in heat after subtracting reference data of BV injections into buffer. The single-binding site model was used to fit the binding isotherms. (**C**) Thermodynamic signatures of BV binding to *Pf* enolase with Gibbs free energy of binding (ΔG; black), enthalpy (ΔH; black and white) and entropy (−TΔS; grey). (**D**) Inhibitory effect of BV on *Pf* enolase activity. (**E**) Non-competitive inhibitory profile of BV. Kinetic activity was assayed in the presence of a fixed dose of BV and increasing concentrations of substrate. First column: negative control [2-PG] = 40 μM (KM value); second column: [2-PG] = 40 μM (KM value) and positive control [BV] = 270 μM (10-fold IC50 value); third column: [2-PG] = 40 μM (KM value) and [BV] = 27 μM (IC50 value); fourth column: [2-PG] = 400 μM (10-fold KM value) and [BV] = 27 μM (IC50 value). Data were analysed from three different experiments.

To assess the ability of *Pf* enolase to bind haem *in vitro*, we expressed recombinant *Pf* enolase in *E. coli* and purified it to homogeneity using a combination of chromatographic methods, including metal chelation and size-exclusion chromatography. We observed a single symmetric peak for a significant pure and folded dimeric 100-KDa protein in the gel filtration column (Fig. 4A). Next, we employed calorimetric analysis using isothermal titration calorimetry (ITC) to evaluate the stoichiometry and energetic components underlying the binding of BV to recombinant purified *Pf* enolase (Fig. 4B). The thermogram suggests an endothermic reaction (Fig. 4B), and the thermodynamic properties indicate that BV binding to *Pf* enolase is more enthalpically favoured (Fig. 4C, represented by larger ΔH bar in the histogram). The evaluated ΔG value of −4.5 Kcal/mol and the binding stoichiometry (n = 1.1) indicate that one BV binds to *Pf* enolase (Fig. 4B).

Thus, we investigated whether BV might affect the catalytic activity of the enzyme. Hence, we evaluated the *Pf* enolase enzymatic activity in the presence of increasing doses of BV (Fig. 4D). BV inhibits the formation of phosphoenolpyruvate (PEP, enolase reaction product) with an IC_50_ value of 27 ± 4 μM. In addition, the effectiveness of BV as a *Pf* enolase inhibitor was evaluated by assessing the mechanism of inhibition. For this purpose, BV concentration was fixed at 27 μM (IC_50_ value), and the *Pf* enolase inhibition was compared in the presence of increasing doses of the substrate (2-phosphoglycerate, 2-PG). Fig. 4E demonstrates that BV inhibitory activity remains unchanged even at concentrations as high as 10-fold of the substrate *K*_M_ value. This result suggests that BV does not act as an active site inhibitor but rather as a non-competitive inhibitor.

### *In silico* docking of BV-*Pf* enolase

**Figure 5:**
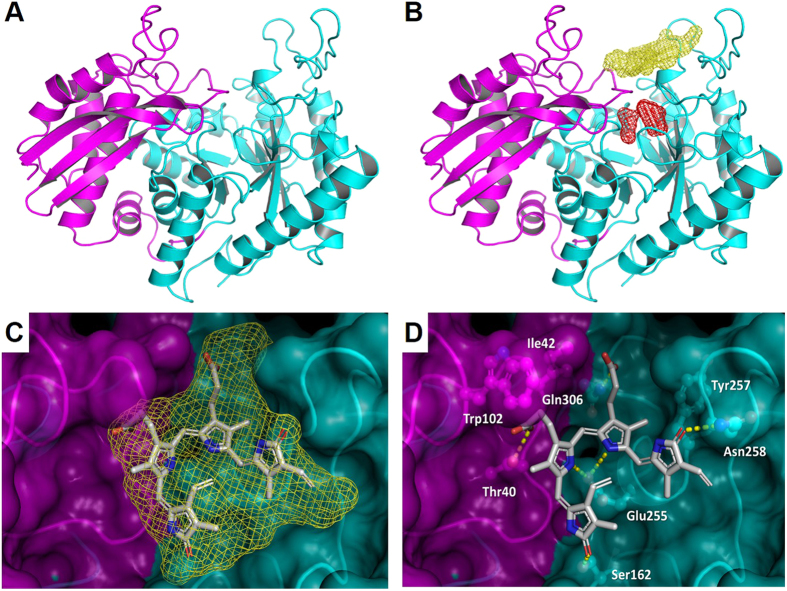
Pf enolase molecular modelling. *Pf* enolase 3D modelling. (**A**) *Pf* enolase homology model (N-terminal domain = magenta; C-terminal domain = cyan). (**B**) *Pf* enolase envelopes for binding cavities identified by SiteHound (catalytic site = red mesh; dimer interface cavity = yellow mesh). (**C**) Modelled binding mode of BV (stick model) into *Pf* enolase putative cavity envelope (yellow mesh). (**D**) Detailed view of BV modelled binding mode (hydrogen bonds = yellow dashes; protein residues = ball and stick model).

Based on the binding data collected and to shed light on the structural determinants underlying BV binding to enolase, molecular modelling approaches were employed to predict potential sites and investigate the BV binding mode. First, a homology-modelling study to predict the *Pf* enolase 3D structure was conducted. A combination of primary sequence and tertiary structure alignments against the known crystal structure of *T. brucei* enolase (PDB ID, 2PTZ) was used to construct a reliable three-dimensional (3D) model of the target protein. The best model comprised a monomeric structure of 439 residues, which exhibited a root-mean-square deviation (RMSD) of 1.6 Å over Cα for 429 equivalent residues of the *T. brucei* homologue. The *Pf* enolase monomer contains an eight fold β/α barrel domain preceded by an *N*-terminal α + β domain (Fig. 5A)^23^. According to the mechanism of inhibition, the BV binding site is somewhere other than the catalytic site. Therefore, SiteHound^24^ was employed to identify putative binding cavities in *P. falciparum* enolase. SiteHound is a web server that identifies ligand-binding sites by computing the interactions between a chemical probe and a protein structure. The top two scored binding cavities correspond to i. the catalytic site and ii. a cavity close to the catalytic site (Fig. 5B). Thus, the second best scored cavity was selected as a putative binding site for the molecular modelling of BV to *Pf* enolase. This putative binding site is located close to the active site and formed by polar and hydrophobic residues from the β/α barrel and α + β domains (Fig. 5B,C).

The proposed binding mode of BV into the putative cavity indicates that the ligand occupies the central region of the putative binding site (Fig. 5D). In this conformation, the ligand establishes attractive polar and hydrophobic contacts with the *Pf* enolase binding site residues. The negatively charged carboxyl groups of BV are hydrogen bonded to the side-chains of Thr40 and Gln306. The electronegative carbonyl substituents of the pyrrolone groups are in a favourable orientation to accept hydrogen bonds from the side-chains of Asn258 and Ser162, whereas the nitrogen atoms of the central pyrrol groups are favourably oriented to donate hydrogen bonds to the side-chain of Glu255. Additionally, non-polar groups of BV make favourable van der Waals contacts with several structural elements of the protein, and some of these groups establish attractive hydrophobic interactions with their macromolecular counterparts that significantly contribute to the complex stability. In particular, the carbon atoms of the carboxyethyl substituent of the pyrrol groups are in van der Waals contact with the side-chains of Trp102 and Ile42; in addition, the 4-methyl substituents of the pyrrol groups are in close contact with the side-chains of Trp102 and Tyr257 (Fig. 5D).

**Figure 6:**
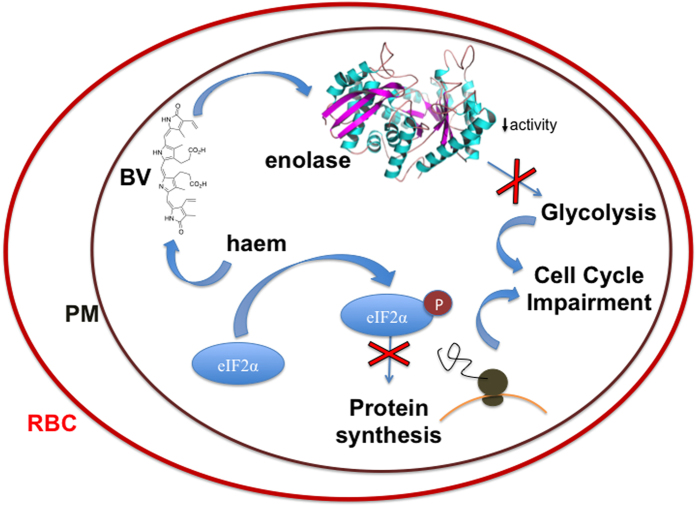
Proposed mechanism for BV reducing the growth of *Plasmodium falciparum*. BV interacts with enolase decreasing its activity and glycolysis and, in turn, cell cycle progression. Moreover, BV and haem modulates phosphorylation of Eukaryotic Initiation Factor 2α (eIF2α). Phosphorylated eIF2α is a stress response factor which acts in the reduction of translation to a basal level and consequently the arrest of the cell cycle of *P. falciparum.* RBC: Red Blood Cell. PM: *Plasmodium falciparum* plasma membrane.

Finally, Fig. 6 depicts a proposed molecular mechanism for BV reducing the growth of *Plasmodium falciparum* cell cycle.

## Discussion

During intraerythrocytic malaria infection, the millimolar amount of haem derivate from host haemoglobin digestion is sequestered as a haemozoin crystal within the digestive vacuole^25^,^26^. Okada 2009^14^ reported the existence of *Pf*HO, which exhibits *in vitro* HO and BVR activity. However, no canonical HO pathway was observed *in vivo* and *in vitro*^17^. The small difference observed in BV concentration in the supernatant of RBC cultures infected and not infected with *P. falciparum* (Fig. 1C) indicates that the parasite does not enzymatically produce or catabolize BV. BR induces oxidative damage and inhibits *P. falciparum* grown in micromolar concentrations^16^. In this study, the concentration of BV detected in *P. falciparum-*infected RBC culture was lower than 100 nM (Fig. 1H). Nevertheless, the levels of BV in human plasma can oscillate up to 40 μM in jaundice patients^27^. As haem catabolism products are toxic to *Plasmodium*, the perception of BV oscillation levels might be an important signal during malaria intraerythrocytic development. Accordingly, the addition of 0.1 and 1 μM BV during 48 hours increased the proportion of mononucleated forms (R and T) and decreased the multinucleated forms (S) without compromising parasitaemia (Fig. 2A). These data suggest that a delay in parasite development might serve as one strategy to address lower increases in BV concentration. Our data on the knockout of *P. berghei* HO indicated that the absence of the gene is lethal to the rodent parasite, suggesting greater vulnerability to haem and its catabolic products. Given that *Pf*HO orthologues are noted in all *Plasmodium* species, HO activity must play a vital role within parasites.

The protein-BV binding assay using zinc acetate in a two-dimensional gel revealed *P. falciparum* enolase as a candidate BV molecular target. Enolase is the eighth enzyme in the glycolytic pathway. Interestingly, based on MS, *Pf* enolase was recently identified in several sub-cellular compartments, including the cytosol, nucleus, cell membrane and food vacuole, despite its lack of an organelle-targeting signal sequence^28^,^29^. This diversity in the sub-cellular localization of enolase suggests that the enzyme may function in cellular recognition/invasion, the formation/development of vacuoles, and transcription^29^ in addition to its role in glycolysis. This phenomenon is known as moonlighting function, and proteins of this class frequently exhibit distinct and physiologically important cellular functions. The multiple functions are not due to protein isoforms; that is, they do not include gene fusions, splicing variants or pleiotropic effects^30^. Enolase is a representative member of the moonlight proteins, acting as a plasminogen receptor^31^, neurotrophic factor^32^, heat-shock protein^33^, hypoxic stress protein^34^, DNA interaction factor^35^, ligand for mosquito gut epithelial receptor^36^, transcription factor in plants^37^ and cancer cells^38^. Another interesting feature of this protein is that enolase is a target for ubiquitination and three forms of the enzyme were identified (MW ~50, 65 and 75 KDa)^39^. In this work, the zinc acetate-binding assay revealed that *Pf* enolase has a MW of 50 KDa, which corresponds to the molecular weight of the non-modified monomer. In fact, the alignment between enolases of different organisms revealed that the proteins have almost the same MW, differing primarily by the presence of a pentapeptide in the apicomplexa phylum. This pentapeptide apparently stabilizes enolase in an active state through the formation of three hydrogen bonds between Ser108 and Leu49, allowing the enzyme to remain active even at high concentrations of ATP^40^. In addition, after RBC invasion by *P. falciparum*, the amount of enolase increases by 15-fold^41^, which is consistent with our quantitative reverse transcriptase-polymerase chain reaction (qRT-PCR) data. These data indicate a sudden increase in expression of this gene in the merozoite stage (Fig. 3C).

As haem and BV delayed the parasite life cycle, we aimed to investigate the molecular mechanism responsible for the observed impairment in the parasite cell cycle. Therefore, we investigated the relationship between the presence of haemin and BV and the phosphorylation state of eIF2α. This factor can be phosphorylated in various stress conditions; thus, protein production is reduced to a basal level in an attempt to circumvent stress. As previously reported^18^,^19^,^20^,^21^, eIF2α kinases possess haem binding sites, and their phosphorylating activity is modulated by haem. We analysed the phosphorylation state of eIF2α by Western blot and reported an increase in eIF2α phosphorylation levels after 5 minutes of treatment followed by a decrease in phospho-eIF2α up to 20 minutes after BV and haem treatment (Fig. 3B). In the same direction, it has been reported that PfPK4 that targets eIF2α as a substrate, has its phosphorylation activity reduced by 60% in the presence of haem at 10 μM^42^. Moreover, Surolia *et al.* 43 had reported a kinase activity in *Plasmodium falciparum* lisates, that resembles HRI activity.

The thermogram data suggest that BV-*Pf* enolase binding is endothermic (Fig. 4A); however, it must be noted that this result might be a consequence of BV dilution. That is, BV dilution is an extremely endothermic reaction and absorbs the heat released from the BV interaction with *Pf* enolase. The evaluated ΔG value of −4.5 Kcal/mol and the binding stoichiometry (n = 1.1) indicate that BV binds favourably to *Pf* enolase. The fact that BV can inhibit *Pf* enolase activity *in vitro* with an IC_50_ value of 27 ± 4 μM (Fig. 4C) is consistent with the observed toxicity in *P. falciparum*-infected RBC culture at 10 μM BV (Fig. 2A). The evaluated BV mechanism of inhibition indicated non-competitive behaviour; that is, *Pf* enolase has an allosteric binding site capable of modulating the enzyme activity. This finding might pave the way for the discovery of new inhibitors as antimalarial candidates. Additionally, *Pf* enolase may act as a sensor for extreme extracellular conditions (*e.g.*, enhanced BV concentration in the cell). It is important to note that the inhibitory activity of *Pf* enolase does not occur at lower BV concentrations (e.g., nM); therefore, another mechanism in addition to the binding to *Pf* enolase must occur for BV to function as a signal molecule.

## Conclusions

In this work, we detected BV in *P. falciparum* culture supernatant and investigated its role in the *P. falciparum* RBC cycle. BV affects the cell cycle of *P. falciparum* by increasing the abundance of the early stages (R and T) and reducing the proportion of S. Thus, we identified *Pf* enolase as a target for BV. The BV binding profile to *Pf* enolase was investigated, and a putative binding site was suggested. On that basis, we propose that BV binding to *Pf* enolase plays a role as a new moonlight function.

Finally, on Fig 6 we propose that the mechanism for the impairment of the cell cycle by BV is associated with a low activity of enolase and alterations to the cell cycle programme due to changes in eIF2α phosphorylation.

## Materials and Methods

### Cell culture, synchronization

The *P. falciparum* strain 3D7 was cultured at 37 °C in RPMI 1640 medium using 10% AB^+^ human serum^44^. Cultures were grown under a 5% CO_2_, 5% O_2_, and 90% N_2_ atmosphere. The culture was synchronized with 5% sorbitol^45^.

### Cloning of *P. falciparum* enolase

*E. coli* Rosetta (DE3) carrying the *P. falciparum* enolase-pETTrx-1a plasmid were cultured at 37 °C with shaking at 150 rev/min in auto-induction medium supplemented with 15 μg/mL kanamycin and 34 μg/mL chloramphenicol for three hours until an OD_600_ of 0.8 was reached. The temperature was then reduced to 20 °C followed by expression for 20 hours. Cells were harvested by centrifugation (30 min; 3,500 g; 4 °C) prior to re-suspension in lysis buffer (buffer A: 50 mM Tris-HCl pH 8.0, 500 mM NaCl, 20 mM imidazole and 10% glycerol) containing 4 mM dithiothreitol and 1 mM phenylmethanesulphonylfluoride. Cells were disrupted by sonication on ice. Insoluble debris was separated by centrifugation (50,000 g; 30 min; 4 °C), and the soluble fraction was filtered and loaded onto a HisTrap HP 5 mL column (GE Healthcare). The His-tagged protein was eluted using a 0 to 1 M imidazole gradient in the same buffer. The excess imidazole was removed by a desalting column (Hi-Trap column - GE Healthcare) followed by TEV protease digestion (1 mg per 20 mg *Pf* enolase). A second passage through the His-Trap column was employed to separate *Pf* enolase from TEV protease, cleaved His-tag-thioredoxin and uncleaved His-tag-thioredoxin *Pf* enolase. The sample containing *Pf* enolase was applied to a Superdex 200, 16/60 column (GE Healthcare), pre-equilibrated in 20 mM Tris-HCl buffer pH 8.0, 200 mM NaCl. The eluted protein was concentrated (10 KDa MWCO Amicon Ultra devices, Millipore) to 10 mg/mL. Protein concentrations were determined spectrophotometrically using a theoretical extinction coefficient of 43,235 mol L^−1 ^cm^−1^ at 280 nm calculated using ProtParam. The high level of protein purity was confirmed by sodium dodecyl sulphate-polyacrylamide gel electrophoresis (SDS-PAGE).

### Sample preparation for HPLC analysis

A 30-mL supernatant sample collected from the desynchronized *P. falciparum* culture and a sample collected from the uninfected RBC culture were concentrated 2-fold by freeze-drying. The resulting suspension was acidified with 200 mL of concentrated hydrochloric acid (HCl_(aq)_36% w/w, Labsynth) and extracted 3 times with 20 mL dichloromethane (Labsynth). The organic solvent was removed under a N_2(g)_ flux and the concentrate resuspended in 1 mL DMSO (Labsynth) and 2 mL methanol (Labsynth).

### HPLC-UV-Vis analysis

HPLC analysis was performed on a Phenomenex Luna 5 m C18 column (250 × 4.60 mm) using a LC 20AT chromatograph equipped with a diode array UV-V is detector (SPD-M20A). The analyses were performed using 5% (v/v) acetonitrile (HPLC/spectro grade, Tedia) with 0.05% (v/v) formic acid (Merck) as solvent A and 100% acetonitrile as solvent B at a flow rate of 0.4 mL/min. Ten minutes after sample injection, a linear gradient of 40 minutes was applied to change solvent A to a mixed solvent A/B (2:8), which was maintained for 20 minutes. The initial condition was restored through a linear acetonitrile gradient (5 minutes).

### HPLC-MS analysis

Mass spectrometry data were obtained in the positive ion mode using an Esquire 3000 Plus mass spectrometer (Bruker Daltonics), coupled with the HPLC instrument. The eluent was the same used in the HPLC analysis with the addition of 2% (v/v) formic acid to solvent A. Samples were introduced into the electrospray source at 70 μL/min. Capillary voltage was set at 4 kV, with a temperature of 250 °C. The nebulizer, dry gas, and dry temperature were set at 20 psi, 7.0 L/min, and 325 °C, respectively.

### Generation of knockout and GFP tagging constructs

Knockout construct transfection was performed on *P. berghei* ANKA strain 2.34 parasites according to a previously published protocol^46^,^47^ with modifications. The *pb HO* knock-out vector was constructed for double crossover homologous recombination in plasmid pBS-DHFR, which contains a *T. gondiidhfr/ts* cassette conferring resistance to pyrimethamine, as described previously^47^,^48^. The knockout construct was generated by inserting the *pbHO* 5′ untranslated region (UTR) region upstream (as Apa1 + HindIII fragment) and the *pbHO* 3′UTR region (as EcoRI + Xba1 fragment) downstream of the *dhfr* cassette. Primers CCCCGGGCCCGGGCGATATGGAATGCACATTTTCTCCTC, N0501 and GGGG*AAGCTT*GCAATCATTTATTCTTTGTAATTG, N0502 were used to generate a 585 bp fragment of the 5′ upstream sequence of *Pbho* from genomic DNA, which was inserted into the *Apa*I and *Hind*III restriction sites upstream of the *dhfr/ts* cassette of pBS-DHFR. Primers CCCC*GAATTC*GAGCCGTAAATGGACACGATGGG (N0503)

and GGGG*TCTAGA*GTCATTTTAAGGTTGGCATTATATTAGC (N0504)

were used to generate a 418 bp fragment from the 3′ flanking region of *Pbho,* which was subsequently inserted downstream of the *dhfr/ts* cassette using *EcoR*I and *Xba*I restriction sites. The final knockout construct was digested with *ApaI* and *XbaI* to release the linearized fragment for transfection. To tag the endogenous locus with GFP, we generated a *pbHO-GFP* construct by single crossover homologous recombination. An 876-bp region coding for the C-terminus of PbHO without the stop codon was inserted in-frame and upstream of the *GFP* sequence in plasmid p277 containing the human *DHFR* cassette and conveying resistance to pyrimethamine, as previously described^49^. The primers used to amplify this fragment are provided below:

CCCCGGTACCGTAGAGATTATATTTACCATCTTGAAG, T1031;

CCCCGGGCCCTTTTTTTATATTTTCAAAATGTTTTGTCAAAATCATC, T1032.

Prior to transfection, the final construct was digested with Bsm1, which cuts the plasmid in the middle of the insert. This cut is optimal for the homologous recombination event. Briefly, electroporated parasites were mixed immediately with 200 μL of reticulocyte-rich blood from a phenylhydrazine (Sigma, UK)-treated naïve mouse and incubated at 37 °C for 30 minutes. Re-invaded parasites were then injected intraperitoneally. From day 1 post infection, pyrimethamine (Sigma, UK) was applied in the drinking water for four days. Mice were monitored for 15 days to allow any slow-growing mutants to reach patency. Drug selection was repeated after passage to a second mouse, and resistant parasites were used for cloning by limiting dilution and genotyping. Transfected *P. berghei* parasites were selected by pyrimethamine pressure in drinking water (7 mg/mL in DMSO dissolved in 500 mL water) or by intra-peritoneal injection (250 μg/100 μL/mouse in DMSO) according to a previously described protocol^50^.

### Real-time qRT-PCR

Total RNA was extracted using Trizol reagent (Invitrogen) according to the manufacturer’s instructions. RNA was quantified using the Nanodrop ND-1000 UV/V is spectrophotometer, and RNA integrity was assessed by electrophoresis in agarose gel. Random-primed RT was performed using 1.0 μg of total RNA according to the Super Script III kit protocol (Invitrogen). The relative transcriptional levels were determined through quantitative PCR (qPCR) with Sybr Green PCR Master Mix (Applied Biosystems) using the 7300 Real-Time PCR System (Applied Biosystems). Real-time data were normalized using the level of expression of seryl-tRNA synthetase and presented as relative expression compared with ring stage parasites. PCR primer sequences targeting enolase mRNA were synthesized as follows: left primer 5′-TTCAGAGCTGCCGTACCATC-3′ and right primer 5′-ACACCCTTTCCTAAGTACCTGC-3′. P-values were determined for the biological triplicates with Student’s *t*-test, using a one-tailed distribution and heteroscedastic variance.

### Protein extraction and western blot

For detection of Phospho-eIF2α,*in vitro P. falciparum*-infected RBCs cultures were synchronized using 5% sorbitol to isolate trophozoites (24 h post invasion) and treated with either 1 μM of BV (Sigma-Aldrich, St Louis, MO, USA, E.U.A.), haem 1 μM (Sigma-Aldrich, St Louis, MO, USA, E.U.A.), and a control sample without any addition. Protein samples of each group were extracted after 1, 5 and 20 minutes of treatment. At each point, the cells were harvested, and the cell pellets were lysed for 60 s in ten volumes of 0.5% saponin prepared in PBS buffer. The parasite pellet was recovered by centrifugation at 8,000 g for 10 min at 4  °C, washed and resuspended in RIPA buffer (25 nM Tris-HCl pH7,4, 150 nM NaCl, 1% NP40, 1% SDS, 2 mM EDTA) with protease inhibitors (1 mM PMSF, 0.01 mM benzamidine, 10 μg /mL aprotinin, 10 μg/mL, leupeptin, 10 μg/mL pepstatin, 10 μg/mL chymostatin, 100 μM sodium orthovanadate, and 20 μM sodium fluoride) and fosfatase inhibitors (β-glicerofosfate, sodium fluoride and sodium pyrofosfate from Sigma-Aldrich, St Louis, MO, USA, E.U.A). Samples were kept on ice for 30 min and particulate material was removed by centrifugation at 14,000 g for 30 min. Protein fractions were separated on 12% SDS-PAGE gels under reducing conditions, transferred to PVDF membranes, blocked with 3% BSA, and probed with anti-Phospho-eIF2α 1:1000 dilution (Cell Signaling) overnight. After washing, the blots were incubated with HRP-conjugated 1:30000 anti IgG rabbit HRP (Ge Healthcare) at a dilution of 1:30000 for 1 h, washed, and visualized using ECL Plus (GE Healthcare). For protein loading control anti-GAPDH antibody was used in 1:5000 dilution (Sigma-Aldrich, St Louis, MO, USA E.U.A.) overnight, and visualized as described above.

### Homology modelling of *P. falciparum* enolase

The 3D model of enolase from *Plasmodium falciparum* was built by homology modelling based on a high-resolution crystal structure of the closest homologous protein available on Protein Data Bank (PDB). The complete amino acid sequence database of the target protein was obtained in FASTA format from the UniProtKB database (Accession N. Q8IJN7). The atomic coordinates of the enolase crystallographic structure from *Trypanosoma brucei* (PDB ID 2PTZ) at 1.65 Å resolution and 62% sequence identity were used as template. The pairwise sequence alignment was performed using ClustalW2^51^, and 10 models were constructed automatically using the MODELLER default parameters (version 9.10)^52^. The lowest global energy (DOPE) model was selected for the validation step, which included inspections of the Ramachandran plot and structural analysis with PROCHECK^53^ and MolProbity^54^. The compatibility of the model with its sequence was assessed by Verify-3D^55^, and the RMSD value between the main-chain atoms of the model and template was calculated by structural superposition of the template and the predicted structure with an optimized algorithm in Pymol 1.2^56^ that performs independent sequence- and structure-based alignment.

### Ligand site prediction of *P. falciparum* enolase

The putative BV binding site was identified by SiteHound, which identifies ligand-binding sites by computing interactions between a chemical probe (*e.g.*, methyl) and a protein structure. The algorithm evaluates the interaction energy between the protein atoms and the molecular probe to identify energetically favourable regions within protein binding cavities. Subsequently, the grid points that exhibit favourable energy values are clustered according to their spatial proximity, and the final output lists the interaction energy clusters corresponding to putative binding sites. The most favourable (lowest energy) binding cavity for BV included residues 38–44, 102–103, 162–163, 253–259, 264–265, 268, 302–308, 312, and 330.

### Molecular docking

Molecular docking and scoring protocols implemented in GOLD 4.1 (Cambridge Crystallographic Data Centre, Cambridge, UK)^57^ were used to investigate the possible binding conformations of the ligands within the *P. falciparum* enolase binding pocket. The *P. falciparum* enolase homology model generated in this work was used in the docking simulations. Hydrogen atoms were added in standard geometry using the Biopolymer module implemented in SYBYL 8.0. Histidine, glutamine, and asparagine residues within the binding site were manually assessed for possible flipped orientation, protonation, and tautomeric states with the Pymol 1.2 (DeLano Scientific, San Carlos, USA) side chain wizard script. The binding site was defined as all the amino acid residues encompassed within a 20-Å radius sphere centred on the Cβ atom of the Ser254 residue. The docking procedures were repeated 30 times for each inhibitor. The implemented GOLD scoring function and visual inspection were employed to select the representative conformation for BV.

### Erythrocyte BV incubation

Erythrocytes infected with synchronized *P. falciparum* (3D7 strain) at the ring stage with 1% parasitaemia were incubated for 48 hours in flat bottom 48-well enzyme-linked immunosorbent assay (ELISA) plates containing different concentrations (0.1, 1 and 10 μM) of BV hydrochloride (Sigma). The controls were infected RBCs with and without DMSO. BV was solubilized in DMSO. After the incubation, the cells were centrifuged, and the pellets were fixed overnight with phosphate buffer solution (PBS) pH 7.4 containing 2% formaldehyde (v/v) (Labsynth). The fixed cells were resuspended in PBS pH 7.4 containing 0.1% Triton-100X (v/v) (Sigma) and 5 nM oxazole yellow homodimer (YOYO-1-labeled DNA, Molecular Probes) and incubated at 37 °C for 30 minutes. Parasitaemia was determined from dot plots (side scatter versus fluorescence) of 10^5^ cells acquired on a FACSCalibur flow cytometer using the CELLQUEST software (Becton Dickinson). Fluorescence was excited with an argon laser at 488 nm, and the fluorescence emission was collected at 520 to 530 nm. Initial gating was performed with unstained, uninfected erythrocytes to account for the erythrocyte autofluorescence.

### Statistical analyses

All results are expressed as the mean ± standard error of the mean (SEM) of at least three individual experiments. A repeated measure one-way analysis of variance (ANOVA) was used for comparisons among larger groups, followed by the Dunnet and Newman-Keuls post-tests. A P-value less than 0.05 was considered indicative of a statistically significant difference. GraphPad Prism software (San Diego, CA, USA) was used for all statistical tests.

## Additional Information

**How to cite this article**: Alves, E. *et al.* Biliverdin targets enolase and eukaryotic initiation factor 2 (eIF2α) to reduce the growth of intraerythrocytic development of the malaria parasite *Plasmodium falciparum. Sci. Rep.* **6**, 22093; doi: 10.1038/srep22093 (2016).

## Supplementary Material

Supplementary Information
