# Supplementary material for: Biliverdin targets enolase and eukaryotic initiation factor 2 (eIF2α) to reduce the growth of intraerythrocytic development of the malaria parasite Plasmodium falciparum
Source: Sci Rep. 2016 Feb 26;6:22093. doi: 10.1038/srep22093 (PMC4768138; doi:10.1038/srep22093)
Supplement: Supplementary Information [file srep22093-s1.doc]

**Supplementary information**

**Biliverdin targets enolase and initiation factor 2 (elf2α) to reduce the growth of intraerythrocytic development of the malaria parasite *Plasmodium falciparum***

**Eduardo Alves,^‡^,*^a,d^* Fernando V. Maluf, ^‡^,*^b^* Vânia B. Bueno,*^c^* Rafael V. C. Guido,*^b^* Glaucius Oliva,*^b^* Maneesh Singh***^a,d^***, Pedro Scarpelli*^a,d^*, Fahyme Costa^a,d^, Robson Sartorello,*^a^* Luiz H. Catalani,*^c^* Declan Brady,*^e^* Rita Tewari*^e^* and Celia R. S. Garcia*^a^,***^*^

*^a^Núcleo de Pesquisa em SinalizaçãoCelular Patógeno-Hospedeiro (NUSCEP), Departamento de Fisiologia, Instituto de Biociências, Universidade de São Paulo.*

*^b^Centro de Pesquisa e Inovação em Biodiversidade e Fármacos, Instituto de Física de São Carlos, Universidade de São Paulo.*

^c^Departamento de Química Fundamental, Instituto de Química, Universidade de São Paulo.

*^d^Departamento de Parasitologia, Instituto de Ciências Biomédicas, Universidade de São Paulo.*

*^e^School of Life Sciences, University of Nottingham, UK*

‡These authors contributed equally to this work.

**Table I**

|  | **Plasmodbacess number** | **Number unique peptide** | **Number Total peptide** | **Function** |
| --- | --- | --- | --- | --- |
| **Spot 1** | Pf3D7_09 | 39 | 136 | Glucose-regulated protein homolog |
|  | Pf14_0517 | 19 | 22 | Peptidase, putative |
|  | PfI0875w | 5 | 18 | Heat shock protein |
|  | Pf3D7_04 | 5 | 5 | Putative uncharacterized protein |
|  | Pf11_0245 | 3 | 3 | Translation elongation factor EF-1, subunit alpha, putative |
|  | Pf08_0054 | 3 | 4 | Heat shock 70 kDa protein |
| **Spot 2** | Pf3D7_09 | 42 | 161 | Glucose-regulated protein  homolog |
|  | PF14_0517 | 18 | 18 | Peptidase, putative |
|  | PF11_0245 | 7 | 7 | Translation elongation factor EF-1 |
|  | PFIT_PFD0095c | 7 | 8 | Plasmodium exported protein (PHISTb), unknown function |
| **Spot 3** | PF08_0054 | 64 | 200 | Heat shock 70 kDa protein |
|  | Pf3D7_09 | 14 | 24 | Glucose-regulated protein homolog |
|  | PF08_0054 | 5 | 7 | Heat shock 70 kDa protein |
| **Spot 4** | PF08_0054 | 91 | 416 | Heat shock 70 kDa protein |
|  | Pf3D7_09 | 9 | 11 | Glucose-regulated protein homolog |
|  | PFC0915w | 5 | 6 | ATP-dependent RNA helicase, putative |
| **Spot 5** | PF10_0155 | 68 | 407 | enolase |
|  | PFL1110c | 3 | 3 | cAMP-dependent protein kinase regulatory subunit |
| **Spot 6** | PFL2215w | 12 | 58 | Actin-1 |
|  | MAL8P1.69 | 7 | 7 | 14-3-3 protein, putative |
|  | PF14_0124 | 3 | 4 | Actin-2 |
| **Spot 7** | PFL2215w | 48 | 185 | Actin-1 |
|  | PF14_0124 | 14 | 16 | Actin-2 |
|  | PFF0435w | 8 | 8 | Ornithine aminotransferase |
|  | PFIT_PF14_0655 | 5 | 5 | Helicase |
|  | PFIT_PFL0185c | 4 | 4 | Nucleosome assembly protein |
|  | MAL8P1.69 | 3 | 3 | 14-3-3 protein, putative |

Table I: Mass spectrometry analyses from binding protein assay in two-dimensional electrophoresis gel of *P. falciparum*-infected RBCs treated with 20 µM BV for 30 minutes The positive spots (1-7) for zinc acetate and comassie stained are showed in Fig. 3A.

The MS was performed by Taplin Biological Mass Spectrometry Facility at Harvard Medical School.
